# Prognostic Significance of Prolonged QTc Interval in Patients on Dialysis: A Retrospective Cohort Study

**DOI:** 10.1002/joa3.70299

**Published:** 2026-02-18

**Authors:** Hoang Nhat Pham, Ramzi Ibrahim, Nathan Giauque, Ahmed K. Mahmoud, Luke Dreher, Luis R. Scott, Chadi Ayoub, Reza Arsanjani, Lisa LeMond, Amitoj Singh, Dan Sorajja

**Affiliations:** ^1^ Department of Medicine University of Arizona Tucson Arizona USA; ^2^ Department of Cardiovascular Medicine Mayo Clinic Phoenix Arizona USA; ^3^ Department of Medicine Boston Medical Center‐Brighton Boston Massachusetts USA; ^4^ Department of Medicine Mayo Clinic Phoenix Arizona USA; ^5^ Sarver Heart Center University of Arizona Tucson Arizona USA

## Abstract

**Background:**

Prolonged QT interval is common in end‐stage renal disease (ESRD) on dialysis, but its long‐term cardiovascular (CV) implications remain unclear.

**Methods:**

Using TriNetX network (2010–2024), we identified adults with ESRD on dialysis and categorized them by QTc status. After 1:1 propensity score matching (*n* = 3428/group), outcomes (hazard ratio [HR]) were assessed using Cox regression.

**Results:**

Prolonged QTc (> 500 ms) was associated with higher risk of all‐cause mortality (HR 1.67; *p* < 0.001), MACEs (HR 1.40; *p* < 0.001), cardiac arrest (HR 1.75; *p* < 0.001), sustained ventricular arrhythmia (HR 1.66; *p* < 0.001), new‐onset atrial fibrillation (HR 1.12; *p* = 0.01), and acute myocardial infarction (HR 2.19; *p* < 0.001).

**Conclusions:**

In ESRD patients on dialysis, prolonged QT interval was independently associated with adverse CV outcomes and mortality.

## Graphical abstract

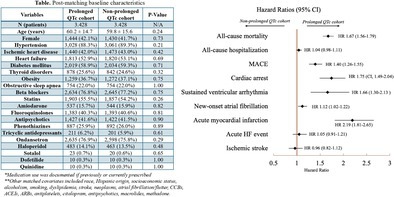

Prolonged QT interval is associated with elevated risk for cardiovascular (CV) events, with the prevalence ranging from 7% to 32% in the general population [1]. In patients with end‐stage renal disease (ESRD) on dialysis, the prevalence of prolonged QTc is substantially higher, often exceeding 40%–65% [2]. Limited data exist on long‐term CV outcomes related to prolonged QT intervals in this patient population. Therefore, we evaluated the prognostic significance of prolonged QT interval in patients on dialysis.

We conducted a retrospective cohort study using the TriNetX Research Network to identify adult patients (≥ 18 years) with ESRD on dialysis between 2010 and 2024. The patients were categorized into two cohorts based on QTc: prolonged QTc versus non‐prolonged QTc cohorts. Prolonged QTc was defined as a QTc > 500 ms, a guideline‐supported threshold associated with a substantially increased risk of malignant ventricular arrhythmias and considered highly abnormal in both sexes [3]. The prolonged QTc cohort included patients with two QTc measurements > 500 ms, taken at least 1 month apart during the dialysis period. QTc was calculated using the Bazett correction formula, which is the default QT correction method within the TriNetX platform. ECGs were obtained during the interdialytic session to minimize confounding from acute electrolyte and volume shifts. Patients with a pacemaker or defibrillator, a diagnosis of long QT syndrome, recent acute myocardial infarction (AMI), or prolonged QRS (> 120 ms) within 1 month of documented prolonged QTc were excluded. Patients were 1:1 propensity score matched (PSM) for demographics, comorbidities, procedures, medications, and laboratory data.

Study outcomes included all‐cause mortality, all‐cause hospitalizations, acute heart failure (HF) events, AMI, cardiac arrest, ischemic stroke, sustained ventricular arrhythmia (SVA), new onset atrial fibrillation (NOAF), and major adverse cardiovascular events (MACEs). MACEs were defined as a composite end point of cardiac death, nonfatal AMI, or stroke. Outcomes were evaluated from the date of the second qualifying QTc measurement to the most recent follow‐up or death. Hazard ratios (HR) and 95% confidence interval (CI) were estimated using Cox regression modeling (Figure 1). Statistical analysis was performed using the TriNetX built‐in function, with statistical significance set at a two‐sided *p* < 0.05. This study does not require Institutional Review Board review nor informed consent as data are de‐identified and in aggregate format, without intervention with human subjects.

**FIGURE 1:**
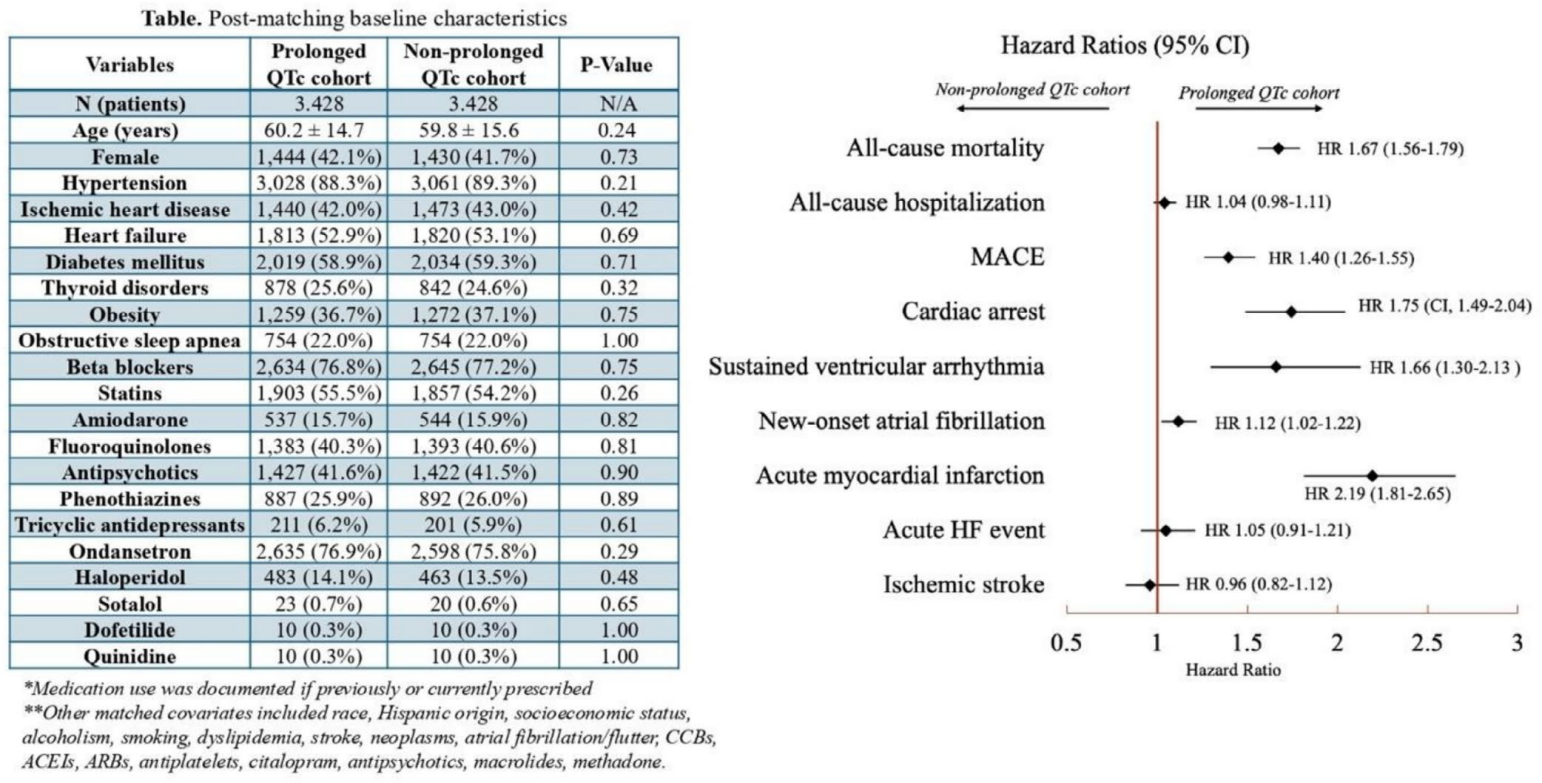
Hazard ratios.

PSM yielded 3428 patients per each cohort with well‐balanced baseline characteristics (mean age 60 years, 42% female). Mean follow‐up duration was 1.5 ± 1.7 and 1.9 ± 1.9 years for prolonged QTc and non‐prolonged QTc cohorts, respectively. Prolonged QTc cohort exhibited a higher hazard for all‐cause mortality (HR 1.67 [95% CI, 1.56–1.79]; *p* < 0.001), MACEs (HR 1.40 [95% CI, 1.26–1.55]; *p* < 0.001), cardiac arrest (HR 1.75 [95% CI, 1.49–2.04]; *p* < 0.001), SVA (HR 1.66 [95% CI, 1.30–2.13]; *p* < 0.001), NOAF (HR 1.12 [95% CI, 1.02–1.22]; *p* = 0.014), and AMI (HR 2.19 [95% CI, 1.81–2.65]; *p* < 0.001). No differences were observed in all‐cause hospitalization (HR 1.04 [95% CI, 0.98–1.11]; *p* = 0.35), acute HF events (HR 1.05 [95% CI, 0.91–1.21]; *p* = 0.52), or ischemic stroke (HR 0.96 [95% CI, 0.82–1.12]; *p* = 0.61).

Our retrospective cohort study is the largest real‐world analysis to evaluate the prognostic implications of a prolonged QT interval in patients with ESRD on dialysis. Prior retrospective analysis examined selected populations and limited outcomes: the Q‐Cohort showed that QTc prolongation in patients on hemodialysis was associated with a more than twofold increased risk of sudden death with adjusted HR 2.10 during 10‐year follow‐up period [4]. Similarly, the TRENDPAD cohort demonstrated that each 10‐ms increase in the baseline QTc conferred a 15% higher risk of all‐cause mortality and MACEs during median follow‐up of 2.2 years in dialysis patients with symptomatic peripheral artery disease [5]. Our study extends these observations by comprehensively evaluating a wide range of clinically relevant outcomes in an real‐world dialysis population, revealing that the presence of a prolonged QT interval was associated with higher risk for all‐cause mortality (67%), MACEs (40%), cardiac arrest (75%), SVA (66%), and NOAF (12%). These findings suggest that QTc prolongation may represent a global marker of cardiovascular vulnerability rather than solely a predictor of sudden death. QT prolongation in ESRD is driven by a combination of modifiable (i.e., electrolyte imbalances, structural cardiac changes, and altered pharmacokinetics) and non‐modifiable factors (i.e., genetic predisposition and age) [2, 6]. QT prolongation tends to increase with dialysis vintage and is particularly unstable during the long interdialytic interval and post‐dialysis period, contributing to elevated risk of sudden cardiac death [6]. Routine assessment of QT interval with targeted risk mitigation strategies may be important in the management of this high‐risk population [1, 6].

Our study was limited due to its inherent retrospective nature using large databases, including potential miscoding and underreporting. The database has no granular, patient‐level data, which restricts our ability to fully understand the underlying mechanisms. For example, duration of dialysis dependency or dialysis vintage, type of access, and etiology of ESRD are not accessible. Furthermore, QTc values in TriNetX are calculated using the Bazett formula, which may overestimate QTc at higher heart rates compared with other correction methods. Despite extensive PSM including adjustment for major comorbidities and QT‐prolonging medications, residual confounding from unmeasured variables may remain. Therefore, causal inferences should be made with caution.

In patients with ESRD on dialysis, a prolonged QT interval is associated with poor cardiovascular outcomes, including all‐cause mortality. Prospective, large‐scale studies are warranted to confirm these findings.

## Funding

This research did not receive any specific grant from funding agencies in the public, commercial, or not‐for‐profit sectors.

## Disclosure

We confirmed that we have not used any AI or AI‐assisted technologies to prepare this work.

## Ethics Statement

The authors have nothing to report.

## Conflicts of Interest

The authors declare no conflicts of interest.

## Data Availability

The data used in this study were obtained from the TriNetX Research Network, a federated health research network that provides access to de‐identified electronic health record data from participating healthcare organizations. The data are not publicly available due to privacy and contractual restrictions. Access to the TriNetX platform is limited to licensed users. Interested researchers may request access to the TriNetX network subject to approval and institutional agreements (https://trinetx.com).
